# Psychiatric History and Trajectories of Cognitive Change Predict Risk of Nursing Home Residence

**DOI:** 10.1093/geroni/igaa057.1511

**Published:** 2020-12-16

**Authors:** Maria Brown, Miriam Mutambudzi

**Affiliations:** 1 Syracuse University, Syracuse, New York, United States; 2 University of Glasgow, Glasgow, Scotland, United Kingdom

## Abstract

Previous research indicates that a history of psychiatric, emotional or nervous problems can affect cognitive function at age 65 and cognitive trajectories over time. To explore the potential impact of this relationship on nursing home use, we applied latent class growth curve models to five waves (1998-2008) of Health and Retirement Study data to identify four classes of cognition trajectories, defined by baseline cognitive function scores (low, medium, high) and rate of change (stable or declining). We then ran survival analyses using HRS years 2008 to 2016 to determine risk of nursing home residence based on psychiatric history and cognition trajectory class. We hypothesized that self-reported history of psychiatric, emotional or nervous problems will be associated with greater risk of nursing home residence, and that self-reported history of psychiatric, emotional and nervous problems will interact with cognition trajectories to predict level of risk of nursing home residence. Results indicate that psychiatric history is independently associated with greater risk of nursing home residence, net the effects of a variety of life course variables in the model. Further, psychiatric history interacts with “low cognition declining” and “medium cognition declining” trajectories to increase the risk of nursing home residence. Evidence of the relationship between psychiatric history, cognitive decline, and nursing home residence can be used to enhance public understanding of the impact psychiatric history has on the long-term care system, and to educate policy-makers and providers about the need for appropriate psychiatric training for staff in community-based and residential long term care programs.

